# Mechanisms of Bone Fragility: From Osteogenesis Imperfecta to Secondary Osteoporosis

**DOI:** 10.3390/ijms22020625

**Published:** 2021-01-10

**Authors:** Ahmed El-Gazzar, Wolfgang Högler

**Affiliations:** Department of Paediatrics and Adolescent Medicine, Johannes Kepler University Linz, Krankenhausstraße 26-30, 4020 Linz, Austria; ahmed.el-gazzar@jku.at

**Keywords:** bone fragility, type I collagen, post-translational modifications, extracellular matrix, osteogenesis imperfecta, Juvenile Paget disease, osteomalacia, osteopetrosis

## Abstract

Bone material strength is determined by several factors, such as bone mass, matrix composition, mineralization, architecture and shape. From a clinical perspective, bone fragility is classified as primary (i.e., genetic and rare) or secondary (i.e., acquired and common) osteoporosis. Understanding the mechanism of rare genetic bone fragility disorders not only advances medical knowledge on rare diseases, it may open doors for drug development for more common disorders (i.e., postmenopausal osteoporosis). In this review, we highlight the main disease mechanisms underlying the development of human bone fragility associated with low bone mass known to date. The pathways we focus on are type I collagen processing, WNT-signaling, TGF-ß signaling, the RANKL-RANK system and the osteocyte mechanosensing pathway. We demonstrate how the discovery of most of these pathways has led to targeted, pathway-specific treatments.

## 1. Introduction

Developing bones consist of cartilaginous joints, the epiphysis, the growth plate cartilage with adjacent osteogenesis and the cortical and cancellous bone mineralized structure. Bone tissue contains three distinct cell types: (i) the osteoblasts, derived from mesenchymal cells, which deposit new bone tissue; (ii) osteoclasts, derived from bone marrow hematopoietic precursor cells, which break down bone matrix; and (iii) osteocytes (former osteoblasts) which orchestrate the activity of osteoblasts and osteoclasts as a response to mechanical strain [1]. The extracellular matrix of bone tissue is composed of inorganic minerals, collagen, water, non-collagenous proteins and lipids. Bone fragility can originate from alterations in all these components, be it the genetic blueprint, mechanical loading, insufficient remodeling at old age, estrogen deficiency or chronic medical conditions affecting bone accrual, structure or composition.

Traditionally, bone fragility is understood as resulting from reduced bone mass, or from defects in bone matrix composition or mineralization. Medical research has unveiled many monogenic bone fragility conditions, yet their mechanisms of disease remain incompletely understood. In fact, they continue holding secrets which may open doors for drug developments in rare and common osteoporosis. In this review, we highlight the main and some exemplary mechanisms underlying human bone fragility associated with reduced bone mass.

Due to word limitations, we are not including groups of conditions associated with bone fragility, such as high bone mass disorders (osteopetrosis) and conditions of bone demineralization (rickets and osteomalacia). In osteopetrosis, fragility is caused by high mineralization density with lack of bone repair [2], and in the demineralization group, fragility is caused by insufficient bone mineral supply or deposition [3].

## 2. Genetic Causes of Bone Fragility

### 2.1. Primary Osteoporosis Affecting Collagen (Osteogenesis Imperfecta)

#### 2.1.1. Clinical Symptoms and Classification

**Figure 1:**
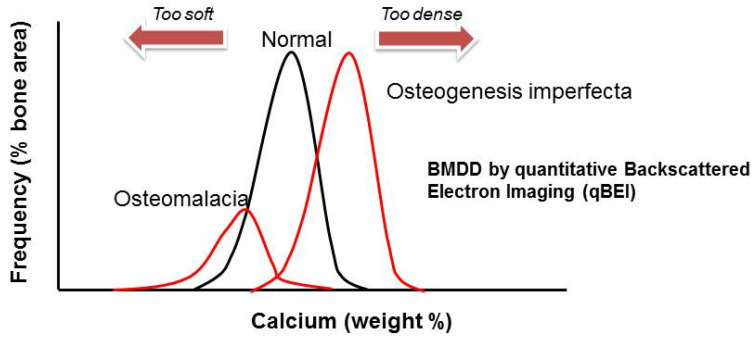
The extremes of bone mineralization density distribution (BMDD): bone tissue in patients with OI has increased mineralization density. This is due to the irregular collagen fibers and their wider spatial distribution which allows more hydroxyapatite deposition. Also depicted is the opposite: low tissue mineralization density in patients with osteomalacia.

Recurrent limb and vertebral fractures are typical for patients with osteogenesis imperfecta (OI). OI, also known as brittle bone disease, is an inherited disorder of connective tissue that has a wide clinical and genetic heterogeneity. OI is a rare disorder, with an incidence of one in 10,000–20,000 births [4]. From a bone material perspective, OI is characterized by low bone mass and increased bone mineralization density, which causes brittleness (Figure 1), recurrent fractures and skeletal deformities, but also extra-skeletal manifestations. The latter include blue sclerae, dentinogenesis imperfecta, joint laxity, hearing loss as well as cranial malformations (i.e., basilar invagination) and pulmonary hypoplasia with reduced lung capacity in severe cases. Depending on clinical severity, mobility is mildly to severely impaired. Fractures are particularly common in childhood but increased fracture risk persists throughout life.

Approximately ~85–90% of OI patients harbor heterozygous mutations in genes encoding type I collagen *COL1A1* [MIM: 120150] or *COL1A2* [MIM: 120160], which are the genes that encode the α1(I) and α2(I) chain of type I collagen, respectively. The remaining ~10–15% harbor mostly recessive mutations in various genes, which were all identified after 2006. While these discoveries have contributed to our understanding of the genetic basis of OI, many molecular disease mechanisms caused by these gene mutations are incompletely understood. Thus far, 24+ genes have been identified to cause OI (Table 1). These include *IFITM5* [5,6] [MIM: 614757], *SERPINF1* [7] [MIM: 172860], *CRTAP* [8] [MIM: 605497], *LEPRE1* [9] [MIM: 610339], *P3H1* [10] [MIM: 610339], *PPIB* [11] [MIM: 123841], *SERPINH1* [12] [MIM: 600943], *FKBP10* [13] [MIM: 607063], *SP7* [14] [MIM: 606633], *BMP1* [15] [MIM: 112264], *TMEM38B* [16] [MIM: 611236], *WNT1* [17,18] [MIM: 164820], *CREB3L1* [19] [MIM: 616215], *SPARC* [20] [MIM: 182120], *FAM46A* [21] [MIM: 611357], *MBTPS2* [22] [MIM: 300294], *MESD* [23] [MIM: 607783], *SEC24D* [24] [MIM: 607186], *CCDC134* [25] [MIM: 618788], *P4HB* [26] [MIM: 176790], *PLOD2* [27] [MIM: 601865], *PLS3* [28] [MIM: 300131] and *KDELR2* [MIM: 609024] [29]. These genes play a critical role in the processing and post-translational modification of type I collagen, the control of osteoblast differentiation or function or the formation of F-actin bundles. Proteins encoded by these genes have their main point of action in the nucleus, the endoplasmic reticulum (ER), the Golgi apparatus, the cytoskeleton or the extracellular matrix. In 1979, prior to any advanced genetic information, David Sillence classified OI into four subtypes according to clinical severity ranging from mild to lethal [30]. This clinical classification is still being used in daily practice, independent of the genetic cause, but a fifth clinical type (OI type 5) has been added which stands out from the other clinical types as it causes hypertrophic callus formation and calcification of interosseous membranes [31]. Classifications remain under debate, and a new classification system has been proposed which groups OI not by simple numbering but by gene cellular function [4,32].

#### 2.1.2. Genetic Classification and Protein Function in OI

**Figure 2:**
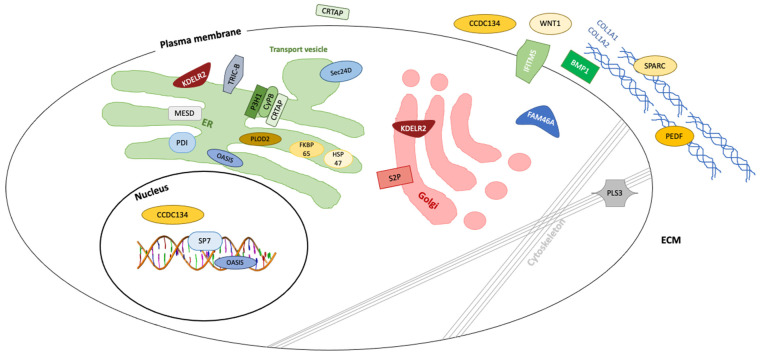
Osteogenesis imperfecta is caused by gene mutations encoding proteins involved in collagen biosynthesis, bone homeostasis and maintenance.

Table 1 contains a list of genes along with their encoded proteins that cause OI or primary osteoporosis, their mode of inheritance and group classification. One recent classification [4] proposes five groups of subtypes for OI (Group A–E). Group A subtypes of OI (Type I–IV, XIII), which are caused by defects in collagen synthesis, structure or processing of COL1A1 and COL1A2, including their C-terminal propeptide cleavage by BMP1. Group B (type VII, VIII, IX and XIV) contains the genes that play a key role in post-translational modification of type I collagen, which are *CRTAP*, *LEPRE1*, *PPIB* and *TMEM38B*. Group C (type X, XI) includes the genes that are responsible for collagen folding or cross linking, which are *SERPINH1*, *FKBP10*, *PLOD2* and *P4HB*. Group D (type V and VI) encompasses the genes *IFITM5* and *SERPINF1* that modify mineralization. Group E (type XII, XV, XVI) involves genes that cause defects in osteoblast differentiation, which are *SP7*, *WNT1* and *CREB3L1*. Figure 2 illustrates the molecular target of all known OI types, their locations in or out of the cells, and which protein products interact with collagen. The figure also depicts the affected cellular and extracellular process, including collagen synthesis, structure and assembly, collagen post-translational modification and processing.

All 24 OI-causing genetic entities have in common that they, by various mechanisms, affect the quality or quantity of type I collagen [4]. Since type I collagen is the main structure protein (94%) of pre-mineralized bone matrix (osteoid), abnormal type I collagen synthesis decreases bone mass and increases susceptibility to fracture. Type I collagen consists of three modified chains that form a triple helical fibril with two identical α1 (I) chains and one structurally similar but genetically different α2 (I) chain. The α chain is characterized by a strict pattern which contains a multiple triple sequence of a Gly (glycine)-X (normally proline)-Y (usually hydroxyproline). Notably, about a third of the residues are prolines that get hydroxylated. Each chain is characterized by amino- and carboxy-propeptides, which are critical in preventing self-assembly of collagen fibrils within the cells.

In the cells, type I collagen is synthesized as a soluble precursor molecule, procollagen with N-terminal and C-terminal propeptides that flank the helical domain. The biosynthesis of type I procollagen is a multistep process that involves an ensemble of proteins for post-translational modifications, folding, transport, secretion and quality control [33]. Procollagen synthesis is started up in the nucleus of collagen producing cells, such as osteoblasts and fibroblasts. The DNA segment is transcribed to precursor RNA that is spliced to mRNA and then transported to the endoplasmic reticulum (rER), via the N-terminal signal peptide, where it is translated to propolypeptide chains termed pro-α chains. In the rER, these propolypeptide chains undergo a series of post-translational modifications described in great detail elsewhere [33,34,35]. The C-terminal propeptide of each chain is attached to the rER membrane and folds into a structure that is stabilized by intra-chain disulfide bonds, which enables the selection and assembly of the correct chains into a triple helix.

Most genes involved in the causation of OI act inside of the ER. However, altered function of ER membrane proteins, such as the TRIC-B calcium channel, encoded by *TMEM38B*, increase ER stress, which affects collagen post-translational modification indirectly, with a variable phenotype [36].

The three modified chains assemble and form procollagen, which is transferred to the Golgi apparatus through larger coat protein II complex (COPII)-vesicles. The COPII machinery is assembled at the ER exit site, under the influence of heat shock protein 47 (HSP47) anchorage to the Src homology 3 (SH3) domain of transport and golgi organization 1 (Tango1) [37]. Following modifications in the Golgi apparatus, procollagen is packed into secretory vesicles which transport it to the extracellular matrix (ECM). Following secretion, specific enzymes, including metalloenzyme ADAMTS2 and bone morphogenetic protein 1 (BMP1), clip off the C-terminal and N-terminal propeptides from the procollagen, which is then termed tropocollagen [38]. Tropocollagens, individual collagen triple helix and the basic structural unit of collagen [39] assemble together to form collagen fibers. In the ECM, secreted acidic cysteine-rich proteins (SPARC) bind to type I collagen in order to form collagen fibrils [20,40].

#### 2.1.3. Pathway-Specific Therapy

To date, there is no pathway-specific therapy that can effectively restore defective collagen processing. However, a number of therapeutic agents can increase overall bone strength and prevent fractures. A combination of anti-resorptive therapy, intensive physiotherapy and muscle training remains the common medical treatment approach in children and adults with OI. Most fractures in OI can be managed by casts or surgery but some leave behind limb deformities, of which many can be corrected by orthopedic rodding surgery.

Currently, bisphosphonates remain the standard anti-resorptive therapy for patients with moderate or severe OI. Bisphosphonates suppress the resorbing activity of osteoclasts and subsequently the typically high bone turnover in OI. Bisphosphonates increase bone mass and mobility in OI and decrease fracture rate in some but not all studies [41]. Notably, RANKL promotes osteoclast differentiation (see Section 2.4). At present, denosumab, a monoclonal antibody against RANKL, which inhibits osteoclastic activity, is being tested in clinical trials in OI patients [42].

Neutralizing antibody against sclerostin (Scl-AB), a molecule produced by osteocytes to inhibit osteoblasts-mediated bone formation via the WNT signaling cascade, increased bone formation rate and bone mass in an OI preclinical mouse model [43].

Recently, preclinical studies demonstrated the potential of MSC transplantation before and after birth for severe types of OI (types II/III, severe type IV); this treatment approach is currently tested in the ongoing multicenter clinical trial boost brittle bones before birth (BOOSTB4) [44]. However, one major hurdle to such a cellular therapy is low engraftment of cells in all skeletal elements.

### 2.2. Primary Osteoporosis Caused by WNT-Signaling Pathway Defects

The Wingless-type mouse mammary tumor virus (MMTV) integration site family 1 (WNT1) protein belongs to a family of 19 secreted signaling glycoproteins. WNTs bind with the frizzled receptor and the co-receptors’ low-density lipoprotein receptor-related protein (LRP)-5 and -6 and activate the β catenin signal transduction pathway in various tissues, including bone [45,46]. This in turn leads to the translocation of β catenin into the nucleus, where it induces the expression of genes that regulate osteoblast differentiation [47]. Thus, the WNT signaling pathway controls mature osteoblast differentiation [48], bone development and bone maintenance [49]. Several groups have shown in patients from different countries that bi-allelic *WNT1* mutations are associated with moderate to severe cases of recessive OI type XV [17,18]. In addition, heterozygous pathogenic mutations in *WNT1* can cause the clinical picture of primary osteoporosis.

Patients that harbor *WNT1* bi-allelic nonsense, missense, splice site substitutions, deletion and frameshift mutations display severe bone fragility, reduced bone mass, multiple fractures and growth delay [18,50]. The structural bone phenotype includes low bone turnover with an imbalance between formation and resorption. This imbalance may be mediated through WNT1 function in osteocytes [51]. Similar to the human nonsense *WNT1* mutation in exon 3 (c.565G > T, p.Glu189*), mice harbor a single nucleotide deletion *wnt1* mutation in exon 3 (c.565delG, p.Glu189Argfs*10) [17,52]. This mouse model shows typical OI features including severe osteopenia, spontaneous fractures, reduced bone strength and impaired matrix mineralization, mimicking the human disease. Notably, bone forming osteoblast function is impaired, while bone resorbing osteoclast function is not changed in this mouse model [53].

*WNT1* mutations arrest the downstream intracellular signaling cascade and nuclear translocation of β catenin and the expression of the regulated genes [17,47]. Loss of function of β catenin results in osteochondroprogenitor cells differentiating into chondrocytes instead of osteoblasts. On the other hand, gain of function of WNT signaling result in enhancement of osteoblast differentiation in vitro [54]. In addition, the WNT signaling cascade in osteocytes plays a critical role in regulating bone cell homeostasis [55]. Mice deficient with β catenin in osteocytes show bone loss [55].

Similar to *WNT1*, biallelic loss of function mutations in its co-receptor *LRP5* cause osteoporosis-pseudoglioma syndrome, which is characterized by severe bone fragility and ocular manifestations [56], and monoallelic *LRP5* mutations, which cause primary, autosomal dominant osteoporosis [57]. Hence, there appears to be a gene dosing effect in defective WNT signaling. Moreover, patients with gain of function and loss of function mutations in the LRP5 have high or low bone mass disorders resulting from constitutive activation or decreased osteoblast activity, respectively [18,58]. This fact further supports the notion of a gene dosing effect and makes elements of the WNT signaling pathway attractive drug targets. Trials have been conducted in adult osteoporosis, which led to the market approval of antibody therapy against sclerostin, an inhibitor of the WNT signaling pathway [59]. Trials in children are awaited.

#### Pathway-Specific Treatment

Sclerostin is a molecule mainly produced by osteocytes and acts as a potent WNT inhibitor. Osteocyte-derived sclerostin controls osteogenic differentiation of precursor cells and bone formation [60]. Lack of osteocyte-derived sclerostin secretion is pathognomonic for the high bone mass disorders sclerosteosis and van Buchem disease and cause excessive bone formation [61,62]. Lessons learned from these forms of osteopetrosis have led to the development of the anti-sclerostin monoclonal antibodies to treat osteoporosis [63,64]. Notably, the sclerostin antibody romosozumab has been approved for osteoporosis treatment in 2019 [63] and has promising potential to treat OI [65]. However, its cardiovascular safety profile will need to be carefully monitored, given the possible role of the Wnt/β-catenin signaling pathway in the progression of atherosclerosis and in vascular calcification [66].

### 2.3. Primary Osteoporosis Caused by Defects in the TGF-β Pathway

Polypeptides in the transforming growth factor β (TGF-β) family are involved in controlling cell activity and metabolism in bone and cartilage tissues. After TGF-β release from the ECM, it interacts with a receptor complex containing type I (TβRI, TGFBR1) and type II (TβRII, TGFBR2) subunits. The highly complex pathway involves intracellular signal transduction through cytoplasmic proteins, belonging to transcription factors from the SMAD family. A variety of heritable skeletal conditions is associated with dysregulated TGF-β signaling, including Camurati-Engelmann disease [MIM: 131300] [67], Marfan syndrome [MIM: 154700] [68] and Loeys-Dietz syndrome [MIM: 613795] [69] but also OI [70]. Bone fragility is a distinct phenotypic feature of Loeys-Dietz syndrome, caused by mutations in SMAD3 (Table 1).

#### Pathway-Specific Treatment

Thus far, murine studies have produced conflicting results, depending on the mouse model used, on whether anti-TGF-β antibodies increase bone mass in OI [70,71]. Apart from antibody-based treatment approaches, TGF-β signaling can also be inhibited using losartan, an angiotensin II type 1 receptor blocker that reduces the expression of TGF-β ligands, receptors, and activators [72]. Treatment with Losartan reduced bone pain and total body BMD in a girl with Camurati-Engelmann syndrome [73]. The TGF-β neutralizing antibody Fresolimumab is currently in clinical trials in children with OI.

### 2.4. Primary Osteoporosis Caused by RANKL/RANK/OPG Defects: TNFRSF11B (Juvenile Paget Disease) and TNFRSF11A (Familial Expansile Osteolysis)

The Receptor Activator of Nuclear Factor Kappa B (RANK, TNFRSF11A) and its ligand RANKL play a key role in osteoclast activation and differentiation [74]. RANKL is a cytokine expressed by osteoblast lineage cells, including osteoblasts and osteocytes. RANKL binds with its cognate receptor RANK on the surface of osteoclast precursors, which activates cell differentiation. Hence, RANKL is essential for formation and activation of osteoclasts. Mice and humans lacking RANKL display complete abrogation of osteoclastogenesis. Osteoblast also express a decoy receptor osteoprotegerin (OPG, TNFRSF11B) that competitively binds with the RANK receptor and inhibits the interaction with RANKL. Hence, osteoblasts and osteocytes regulate activation and differentiation of osteoclasts by the RANKL-RANK axis signaling pathway [74,75].

A recessive deletion mutation in *TNFRSF11B* encoding the decoy receptor OPG results in unopposed RANK activation and permanently elevated bone turnover, a condition called Juvenile Paget’s disease (JPD; [MIM: 602080]) [76]. JPD is a rare autosomal recessive disease which develops during infancy and early childhood, and worsens in adolescence with pain from debilitating fractures and deformities caused by highly accelerated bone turnover of the entire skeleton [77]. JPD also has extra-skeletal manifestations, including bowing deformities and fractures, contractures as well as short stature [78]. Patients demonstrate histopathological evidence of high bone turnover and weak, disorganized woven bone [79,80].

Dominant gain of function mutations in *TNFRSF11A* cause familial expansile osteolysis [FEO; MIM: 174810], leading to permanent activation of RANK. The ensuing progressive osteoclastic resorption is associated with medullar expansion and severe pain, disabling deformities and pathological fractures. Characteristically, FEO is accompanied by deafness and loss of dentition as a result of middle ear and jaw bone abnormalities, and variably raised serum alkaline phosphatase levels. FEO cases present with osteolytic lesions in long bones, whereas JPD patients tend to present with trunk and skull lesions [81].

#### Pathway-Specific Treatment

Denosumab (Denosumab), a monoclonal antibody that binds human RANKL has shown therapeutic efficacy in patients with JPD. Adult JPD patients with a milder phenotype treated with Denosumab demonstrated clinical and biochemical remission of the skeletal disease [82]. In a child with JPD with a severe phenotype, Denosumab reduced bone pain and bone turnover, better than bisphosphonate treatment, and was accompanied by improved audiological tests in a short-term study [83]. Notably, severe hypocalcemia from oversuppression of bone resorption has been observed after Denosumab administration in this patient. High-dose Denosumab has received a license for the treatment of Giant-cell tumors of bone (GCTB), which have osteoclast-like giant cells [84]. However, following cessation of treatment, the release of bone (over)suppression can cause severe hypercalcemia, particularly in young patients [85]. More clinical trials are required to test the safety and efficacy of Denosumab in children with bone fragility.

The imbalance in the RANKL-RANK signaling pathway is a feature in many rare metabolic bone diseases, including JPD, fibrous dysplasia, Hajdu Cheney syndrome and Langerhans cell histiocytosis [86,87].

### 2.5. Bone Fragility in Hajdu Cheney Syndrome

In bone, NOTCH signaling is involved in the regulation of bone formation and bone resorption [88]. In vitro studies have suggest that the NOTCH signaling cascade modulates signaling downstream of RANK, hence activating NOTCH2 enhances osteoclasts maturation [89]. NOTCH2 plays a key role in skeletal development [90]. Homozygous deletion of mouse *Notch2* results in early embryonic lethality [91]. Heterozygous gain of function mutations in *NOTCH2* lead to Hajdu-Cheney syndrome (HCS) [92]. HCS [MIM: 102500] is a rare autosomal dominant disease characterized by severe osteoporosis associated with craniofacial dysmorphism, acroosteolysis and Wormian bones [93]. Nonsense or short deletion mutations in *NOTCH2* exon 34 (the last exon) result in an early termination upstream of the Proline-Glutamic acid-Serine-Threonine (PEST) domain, at the end of the protein, which is required for the NOTCH2 receptor ubiquitination and degradation [94]. Hence, these mutations produce a truncated protein lacking the proteolytic degradation domain of NOTCH2 receptors, leading to sustained NOTCH2 activation with increased osteoclastogenesis [95]. Histomorphometric analysis in affected individuals demonstrate increased bone resorption, increased heterogeneity of mineralization and woven bone. Typical features are reduced cortical thickness and low bone mass. Given the increased osteoclast numbers and turnover, affected individuals respond well to bisphosphonate therapy [96].

## 3. Acquired Causes of Bone Fragility

### 3.1. Immobility-Induced Osteoporosis Caused by the Osteocyte Biomechanic Sensing Mechanism

Muscle force drives bone strength. During immobilization, lack of muscle tension results in reduced bone loading, particularly in bones in the trunk and lower extremity, which leads to loss of bone mass, or inadequate bone accrual with typically slender long bones [97]. In humans with partial or complete immobility, the functional muscle-bone unit adapts to a lower steady state, so osteoporosis may show little or no sign of progression and pathological limb fractures usually occur due to external forces [98], and vertebral fractures are rare. Given the large number of disabled and immobilized people, this form of osteoporosis is probably the most common worldwide.

The mechanism of disuse osteoporosis relates to the ability of bones for mechanosensing. Osteocytes make up >90% of all bone cells and the healthy human skeleton contains around 42 billion osteocytes which live for decades [99], whereas the bone-forming osteoblasts and bone-resorbing osteoclasts make up around 4–6% and 1–2%, respectively, and live only for a few days to weeks [100].

Osteocytes form an extensive cellular network of sensory cells mediating the effects of mechanical loading of bone, which triggers interstitial fluid flow through the lacuna-canalicular system [101]. Osteocytes interact with the ECM through their cell membrane proteins integrin and vinculin and through transverse tethering elements that anchor and center osteocytes to the canalicular wall [102]. Mechanical forces alter canalicular fluid flow and activate the ERK signaling cascade with attenuation of osteocyte apoptosis [103]. Conversely, disuse induces osteocyte apoptosis [104,105].

The cellular and molecular mechanisms by which reduced mechanical forces induced osteocyte apoptosis have not been fully elucidated. Osteocytes produce various signaling proteins, including sclerostin, WNT1, RANKL and vascular endothelial growth factor (VEGF) [106]. Osteocyte is the main source of sclerostin, a potent Wnt inhibitor, which controls osteogenic differentiation of precursor cells and bone formation [60]. Genetic defects in the production of these osteocytic proteins causes rare bone diseases [45,61]. Acquired disruption may have similar consequences. Long-term unloading, for example in astronauts during space travel or long-term bed rest, increases *SOST* (sclerostin gene) expression in osteocytes, hence increasing sclerostin [107,108]. Mechanical loading reduces osteocyte-mediated sclerostin secretion, whereas proinflammatory cytokines also promote its production [109].

#### Pathway-Specific Treatment

To date, studies using sclerostin antibodies in disuse osteoporosis have not been conducted. The obvious treatment of disuse osteoporosis would of course be physical activity, but in many cases, this is not possible. Whole body vibration therapy is an alternative which uses mechanical stimuli to target osteocyte mechanosensing [110]. Such mechanical stimuli prompt osteocytes to release nitric oxide (NO), prostaglandins (PGs) and ATP, which regulate various signaling cascade including interleukin-6 (IL-6), RANKL/OPG, Wnt/β-catenin and calcium signaling pathways [111]. Mechanical loading-mediated calcium oscillation results in the release of extracellular vesicles from osteocytes and directs bone regeneration [112]. Furthermore, mechanical loading promotes calcium oscillation in osteocytes and activates the release of NO [113], prostaglandin E2 (PGE2) [114], matrix extracellular phosphoglycoprotein (MEPE), insulin-like growth factor-1 (IGF-1) [115] and β-catenin [116]. In addition, mechanical stimulation of chicken and canine bone also increased PGE2 [117,118], which is considered an apoptosis inhibitor [119]. Thus, the decrease of NO and PGE2 may be involved in the mechanism by which reduced mechanical forces mediate osteocyte apoptosis. Further studies are needed in this area.

### 3.2. Cytokine-Induced Osteoporosis in Leukemia/Cancer or Chronic Inflammatory Conditions via RANKL Activation

The imbalance between bone resorption and formation occurs not just in osteopetrosis or osteoporosis, but particularly in malignant and inflammatory bone diseases such as acute leukemia of rheumatoid arthritis [120]. In these conditions, a large number of cytokines are produced by tumor or inflammatory cells. Cytokines activate the RANKL-RANK system [121]. Factors that can activate bone resorption, including PTHrP, IL-1, IL-11, IL-17 and TNF-α [122], and induce RANKL expression on osteoblasts, which in turn binds RANK on osteoclasts progenitors, promoting preosteoclast differentiation into osteoclasts. Furthermore, RANKL plays a key role in the survival and function of osteoclasts. RANKL is produced as a membrane-bound protein and also as a soluble trimeric protein [123]. The decoy receptor OPG which competes with RANKL for binding the RANK receptor, is induced by estrogen and transforming growth factor β (TGF-β) [124]. In healthy people, the relative levels of OPG and RANKL are tightly controlled. In a pathological condition, such as postmenopausal osteoporosis, reduced estrogen levels cause decreased OPG, and consequently, increased RANKL, leading to increased osteoclastic bone resorption and bone loss [125]. Recently, a new receptor for RANKL known as leucine-rich repeat G protein coupled receptor 4 (LGR4) has been identified [126]. LGR4 is also expressed on osteoclasts and acts as negative regulator for osteoclast differentiation. Hence, LGR4 and OPG inhibit the RANKL-RANK signaling cascade. Moreover, tumor cells can enhance osteoclasts-mediated osteolysis by several mechanisms, including expression of RANKL. Tumor cells can also express growth factors such as PTHrP that can induce expression of RANKL on osteoblasts, which in turn result in differentiation of multinucleated osteoclasts from myeloid precursors [127]. Consequently, mature osteoclasts resorb bone matrix, permitting tumor cells to grow and migrate within the tissues.

Taken together, agents inhibiting the RANKL-RANK system overcome the decreased remodeling efficiency (more resorption than formation). The monoclonal RANKL antibody Denosumab inhibits osteoclasts and has been approved for the therapy of various bone-associated diseases including postmenopausal osteoporosis [74].

### 3.3. Steroid-Induced Osteoporosis (Osteotoxic Glucocorticoid Medication)

Steroid-induced, or glucocorticoid-induced, osteoporosis (GIOP) is a frequent cause of secondary osteoporosis. The frequency of GIOP in the population ranges from 0.5–1% [128,129]. Glucocorticoids are prescribed widely for inflammatory and immune diseases [130] which themselves, via cytokines, increase osteoclastogenesis.

Glucocorticoids increase the risk of vertebral and non-vertebral fractures [131] via direct and indirect cellular effects. Direct effects of glucocorticoids include (i) impaired function and decreased number of bone forming osteoblasts as well as osteocytes. Stimulation of caspase 3-mediated apoptosis of osteoblasts and osteocytes results in reduced bone mass and impaired bone microstructure. Additionally, reduction of Wnt signaling by Dickkopf-1 (Dkk-1) and sclerostin suppresses the stabilization of β-catenin, and in turn, leads to the inhibition of osteoblastogenesis. Furthermore, increased expression of the peroxisome proliferation activated receptor γ2 (PPAR-γ2) signaling cascade, leads to the activation of adipogenesis in bone tissue [132]. (ii) Moreover, glucocorticoids promote osteoclastogenesis by increasing expression of RANKL and macrophage colony-stimulating factor (M-CSF) and decreasing expression of OPG in osteoblasts and osteocytes. Indirect effects include lack of synthesis of sex steroids; mediating muscle mass loss; inhibiting IGF-1 and its binding protein; and decreasing calcium absorption by renal and intestinal tissue [132]. Treatment of GIOP is not specifically inhibiting the osteotoxicity of glucocorticoids; instead, a number of drugs used for common osteoporosis have been trialed [133].

## 4. Conclusions

The genetic spectrum of primary osteoporosis has expanded massively in recent years. Thus far, at least 24 genes have been identified to cause OI. Mechanistic studies in vitro and preclinical mouse models have demonstrated defects in type I collagen processing and crosslinking, post-translational modifications, folding, procollagen transport from rough ER to the Golgi or collagen secretion and structure. Notably, some forms of OI such as *IFITM5* or *SERPINF1*, are associated with impaired mineralization, and *SP7* or *WNT1* are associated with impaired osteoblast differentiation. Rare fragility conditions, outside the collagen processing pathway, have received less attention, such as those affecting the WNT-LRP5 signaling pathway, the RANKL-RANK system and the NOTCH2 signaling pathway. The understanding of these pathways through the study of rare bone diseases has led to the development of specific therapeutics agents such as denosumab and anti-sclerostin antibodies for the treatment of common osteoporosis. To date, many rare fragility disorders remain incompletely understood and hence drug targets still remain undiscovered for similar future drug development. To date, acquired bone fragility conditions (immobility-, cytokine and glucocorticoid-induced as well as postmenopausal osteoporosis) are far more common and new, pathway-specific treatments are still needed.

## Figures and Tables

**Table 1:** Bone fragility conditions with low bone mass: inheritance, gene defects, protein function and clinical characteristics, grouped by genes causing osteogenesis imperfecta, other primary osteoporosis and osteolysis.

Condition	OMIM	Inheritance	Gene	Mutation	Protein	Bone Pathway	Symptoms
Osteogenesis imperfecta and primary osteoporosis	166200	AD	*COL1A1* *COL1A2*	Loss of function	Collagen α1(I) chain Collagen α2(I) chain	Collagen synthesis	OI 1 (clinical type I, mild)
	166210						OI 2 (clinical type II, perinatal lethal)
	259420						OI 3 (clinical type III, severe)
	166220						OI 4 (clinical type IV, moderate)
	610967	AD	*IFITM5*	Gain of function	Interferon-induced Transmembrane protein 5 (BRIL)	Mineralization	OI 5 (clinical types V; and III in atypical OI 6)
	613982	AR	*SERPINF 1*	Loss of function	Pigment epithelium-derived factor (PEDF)	Mineralization	OI 6 (clinical type III)
	610854	AR	*CRTAP*	Loss of function	Cartilage-associated protein (CRTAP)	Collagen modification	OI 7 (clinical types II, III, IV)
	610915	AR	*LEPRE1* *(P3H1)*	Loss of function	Leucine proline enriched proteoglycan1/Prolyl 3-hydroxylase 1 (P3H1)	Collagen modification	OI 8 (clinical types II, III)
	259440	AR	*PPIB*	Loss of function	Cyclophilin B (CyPB)	Collagen modification	OI 9 (clinical types II, III)
	613848	AR	*SERPINH1*	Loss of function	Serpin peptidase inhibitor, clade H, member 1/heat shock protein 47	Collagen folding and cross-linking	OI 10 (clinical type III)
	610968	AR	*FKBP10*	Loss of function	Peptidyl-prolyl cis-transisomerase FKBP10	Collagen folding and cross-linking	OI 11 (clinical types III, IV)
	259450	AR					Bruck Syndrome Type 1 (BS1)
	613849	AR	SP7	Loss of function	Zinc-finger transcription factor, Osterix	Osteoblast differentiation and maturation	OI 12 (clinical type IV)
	112264	AR	*BMP1*	Loss of function	Bone morphogenic protein1/procollagen C proteinase	Collagen processing	OI 13 (clinical Type III)
	615066	AR	*TMEM38B*	Loss of function	Trimeric intracellular cation channel B (TRIC-B)	ER calcium flux	OI 14 (clinical type I, III, IV)
	615220	AR	*WNT1*	Loss of function	Wingless-type MMTV integration site family, member 1	WNT signaling	OI 15 (clinical type III, IV)
		AD					Primary osteoporosis
	616229	AR	*CREB3L1*	Loss of function	Old astrocyte specifically induced substance (OASIS)	ER UPR response, ER-Golgi trafficking	OI 16 (clinical type III)
		AD					OI 16 (clinical type I)
	616507	AR	*SPARC*	Loss of function	Secreted protein, acidic, cysteine-rich (SPARC, or osteonectin)	Procollagen processing and extracellular assembly	OI 17 (clinical type III, IV)
	617952	AR	*TENT5A (FAM46A)*	Loss of function	Terminal nucleotidyltransferase 46, Member A (FAM46A)	BMP signaling	OI 18 (clinical type III), overlap with Stuve-Wiedemann syndrome
	601559						
	301014	XR	*MBTPS2*	Loss of function	Site 2 protease (S2P)	Golgi Regulated intramembrane proteolysis	OI 19 (clinical type III, IV)
	607782	AR	*MESD*	Loss of function	Mesoderm development LRP chaperon	WNT signaling	OI 20 (clinical type III)
	607186	AR	*SEC24D*	Loss of function	SEC24D	ER COPII Transport of procollagen	OI (clinical type III), overlap with
							Cole-Carpenter Syndrome 2
	618788	AR	*CCDC134*	Loss of function	Coiled-coil domain containing 134	MAPK pathway	OI (clinical type III)
	609024	AR	*KDELR2*	Loss of function	KDEL endoplasmic reticulum protein retention receptor 2	Regulate the trafficking of proteins between the Golgi apparatus and the ER	OI (clinical type IIB/III)
Other Primary Osteoporosis	259770	AR	*LRP5*	Loss of function	Low density lipoprotein receptor 5 (LRP5)	WNT signaling	Osteoporosis pseudoglioma syndrome
	166710	AD					Primary osteoporosis
	300910	XL	*PLS3*	Loss of function	Plastin 3	Formation of F-actin bundles	Primary osteoporosis
	609220	AR	*PLOD2*	Loss of function	Telopeptide lysyl hydroxylase	Collagen crosslinking	Bruck Syndrome 2 (BS2)
	126550	AD	*SGMS2*	Loss of function	Phosphatidylcholine:ceramide cholinephosphotransferase 2	Mineralization	Calvarial doughnut lesions with bone fragility without (CDL) or with spondylometaphyseal dysplasia (CDLSMD)
	112240	AD	*P4HB*	Loss of function	Protein disulfide-isomerase	Catalyzes rearrangement of disulfid bonds	Cole-Carpenter syndrome 1
	605822	AR	*XYLT2*	Loss of function	Xylosyltransferase 2	Proteoglycan biosynthesis	Spondylo-ocular dysplasia
	166260	AD	*ANO5*	Loss of function	Anoctamin-5	Unclear (chloride channel)	Gnathodiaphyseal dysplasia
	231070	AR	*GORAB*	Loss of function	RAB6-interacting golgin	Unclear	Geroderma osteodysplasticum
	612940	AR	*PYCR1*	Loss of function	Pyrroline-5-carboxylate reductase 1, mitochondrial	Unclear (Prolin biosynthesis)	Cutis laxa (ARCL2B)
	182250	AD	*IFIH1*	Gain of function	Interferon-induced helicase C domain-containing protein 1	Unclear (Antiviral innate immunity)	Singleton-Mertin dysplasia Type 1
	616298	AD	*DDX58*	Gain of function	Antiviral innate immune response receptor RIG-I	Unclear (antiviral innate immunity)	Singleton-Mertin dysplasia Type 2
	616866	AR	*TRIP4*	Loss of function	Activating signal cointegrator 1	Unclear (transcription coactivator)	Spinal muscular atrophy with congenital bone fractures-1 (SMABF1)
	616867	AR	*ASCC1*	Loss of function	Activating signal cointegrator 1 complex subunit 1	Unclear (DNA damage repair)	Spinal muscular atrophy with congenital bone fractures-2 (SMABF2)
	603109	AD	*SMAD3*	Loss of function	Smad family member 3	TGF-ß pathway	Loeys-Dietz syndrome
Osteolysis Group	174810 602080	AD	*TNFRSF11A*	Gain of function	Tumor necrosis factor receptor superfamily member 11A	RANK overactivation	Familial expansile osteolysis (FEO) Juvenile Paget’s Disease (PDB2)
	239000	AR	*TNFRSF11B*	Loss of function	Tumor necrosis factor receptor superfamily member 11B	OPG deficiency with Increased RANKL-mediated osteoclastogenesis	Juvenile Paget’s Disease (PDB5)
	259600	AR	*MMP2*	Loss of function	Matrix metalloproteinase 2	Unclear (collagenolysis)	Multicentric osteolysis, nodulosis and arthropathy (MANO)
	277950		*MMP14*		Matrix metalloproteinase 14		
	102500	AD	*NOTCH2*	Gain of function	Neurogenic locus notch homolog protein 2	Regulate cell fate; osteoblast and osteoclast function	Hajdu-Cheney Syndrome

## Data Availability

Not applicable.

## Literature Search Strategy

In this review, both open access scholarly sources and subscription-based journals were considered for the study. A computerized literature search was performed on PubMed, not limited by publication time. Articles were selected if they had been published as full journal articles in English. Articles could be either original research papers, reviews or book chapter discussing the relevant topics OI and osteoporosis. We used the terms in combination with appropriate logical connectors; such as “Type I collagen- osteogenesis imperfecta,” “glucocorticoid-induced osteoporosis,” “steroid- induced osteoporosis,” “glucocorticoids,” “osteoporosis,” “RANK with osteoporosis,” “RANKL with osteoporosis” and “OPG with osteoporosis,” last update: 19 October 2020. The literature search was extended by ‘hand searching’ the reference list of the retrieved articles. Automatic alerts, until the first submission of the review, were activated in Pubmed ‘my NCBI’ to include relevant publications after the primary search.
